# Moderate-intensity statin therapy seems ineffective in primary cardiovascular prevention in patients with type 2 diabetes complicated by nephropathy. A multicenter prospective 8 years follow up study

**DOI:** 10.1186/s12933-016-0463-9

**Published:** 2016-10-13

**Authors:** Ferdinando Carlo Sasso, Nadia Lascar, Antonella Ascione, Ornella Carbonara, Luca De Nicola, Roberto Minutolo, Teresa Salvatore, Maria Rosaria Rizzo, Plinio Cirillo, Giuseppe Paolisso, Raffaele Marfella, on behalf of NID-2 study group

**Affiliations:** 1 Department of Internal and Experimental Medicine ‘‘Magrassi-Lanzara’’, Second University of Naples, Naples, Italy; 2 School of Life and Health Sciences, Aston University, Birmingham, UK; 3 Unit of Nephrology, Second University of Naples, Naples, Italy; 4 Department of Geriatrics and Metabolic Diseases, Second University of Naples, Naples, Italy; 5 Department of Advanced Biomedical Science, University of Naples Federico II, Naples, Italy

**Keywords:** CVD, Primary prevention, Statin, Diabetes, Nephropathy

## Abstract

**Background:**

Although numerous studies and metanalysis have shown the beneficial effect of statin therapy in CVD secondary prevention, there is still controversy such the use of statins for primary CVD prevention in patients with DM. The purpose of this study was to evaluate the occurrence of total major adverse cardio-vascular events (MACE) in a cohort of patients with type 2 diabetes complicated by nephropathy treated with statins, in order to verify real life effect of statin on CVD primary prevention.

**Methods:**

We conducted an observational prospective multicenter study on 564 patients with type 2 diabetic nephropathy free of cardiovascular disease attending 21 national outpatient diabetes clinics and followed them up for 8 years. 169 of them were treated with statins (group A) while 395 were not on statins (group B).

**Results:**

Notably, none of the patients was treated with a high-intensity statin therapy according to last ADA position statement. Total MACE occurred in 32 patients from group A and in 68 patients from group B. Fatal MACE occurred in 13 patients from group A and in 30 from group B; nonfatal MACE occurred in 19 patients from group A and in 38 patients from group B. The analysis of the Kaplan–Meier survival curves showed a not statistically significant difference in the incidence of total (p 0.758), fatal (p 0.474) and nonfatal (p 0.812) MACE between the two groups. HbA1c only showed a significant difference in the incidence of MACE between the two groups (HR 1.201, CI 1.041–1.387, p 0.012).

**Conclusions:**

These findings suggest that, in a real clinical setting, moderate-intensity statin treatment is ineffective in cardiovascular primary prevention for patients with diabetic nephropathy.

*Trial registration* ClinicalTrials.gov Identifier NCT00535925. Date of registration: September 24, 2007, retrospectively registered

## Background

There is a strong correlation between diabetes mellitus (DM) and cardiovascular disease (CVD). Cardiovascular complications account for 50–80 % of early deaths in diabetes patients. A large Danish study [1] showed that patients with DM and no prior myocardial infarction (MI) exhibited a 20 % cardiovascular death risk over a 7 years period, which is comparable to those without diabetes but prior MI. Moreover, people with type-2 diabetes (T2DM) are two to four times more likely to develop coronary artery disease, cerebrovascular disease and peripheral vascular disease [2, 3], compared to the general population. According to the World Health Organization, CVDs are the leading cause of death globally [4] and accelerated atherosclerosis is the cause of the majority of cardiovascular events [5]. The most recent guidelines of the European Society of Cardiology (ESC) state that people with type 2 diabetes are automatically at high CV risk. Moreover subjects with diabetes and target organ damage such as proteinuria, are at very high CV risk [6]. Therefore, diabetic nephropathy, which represents a major form of chronic kidney disease and a leading cause of end-stage renal disease, is also a risk factor for CVD [7]. The underlying pathogenic mechanism that links diabetic nephropathy to a high risk of CVD remains unclear. This is probably associated to endothelial damage through inflammation and oxidative stress. Moreover advanced glycation end products may play a role in the development and progression of atherosclerosis in patients with diabetic nephropathy [8]. Several studies have shown that diabetic nephropathy is a prognostic indicator of early mortality from CVD independently on the mechanisms involved in its development [9].

In people with DM, intracellular hyperglycaemia has been shown to lead to the generation of advanced glycation end-products and reactive oxygen species which might activate a number of pro-inflammatory pathways, induce endothelial dysfunction, cause long-lasting modifications of the arterial wall and enhance insulin resistance [10–12]. These pathways contribute to the development of the atherogenic dyslipidaemia in diabetes, characterized by high serum triglycerides (TG), high small dense low-density lipoprotein cholesterol (LDL-C) levels and low high-density lipoprotein cholesterol (HDL-C) levels [13, 14]. Hyperlipidaemia is a significant modifiable risk factor that can be targeted. With this regard the latest statement of the American Diabetes Association (ADA) recommends lifestyle intervention to improve the lipid profile in patients with DM and initiate statin therapy for patients with overt CVD (level of evidence A), those aged 40–75 years regardless of additional CV risk factors (level of evidence A) and those aged below 40 years with additional risk factors (level of evidence C) [15]. Although numerous studies and metanalysis have shown the beneficial effect of statin therapy in CVD secondary prevention [16–19], there is still controversy such the use of statins for primary CVD prevention [20–23] in patients with DM.

Moreover few clinical trials showed uncertainty around statin therapy in people with DM as the effects on particular CV outcomes could be influenced by other factors, such as blood pressure and glycaemic control [24]. There is a lack of evidence in the role of statin treatment of selected categories of diabetic patients at very high CV risk, such as those affected by diabetic nephropathy. There is also the need for awareness of risks as well as benefits around the use of this class of medications for CV primary prevention, especially their potential for adverse effects [25] and their safety in older patients [26].

## Methods

### Aim, design and setting of the study

We evaluated the use of statins in a cohort of patients with T2DM complicated by nephropathy, in order to verify the effects for CVD primary prevention in this high risk population.

We conducted an observational prospective multicenter study on a subgroup of type 2 diabetes patients from the NID-2 study cohort [27], selecting for the specific purpose of the current study only patients free of CVD at baseline. CVD included: coronary artery disease (MI, angioplasty, coronary bypass graft), cerebrovascular disease (TIA, stroke) and peripheral vascular disease (occlusive arteries disease, revascularisation, major amputation).

The NID-2 study recruited 847 patients with type 2 diabetes at very high CV risk, complicated by diabetic nephropathy III–IV stage (diagnosed by clinical criteria, i.e., micro/macro-albuminuria and moderate/severe diabetic retinopathy) from 21 clinics of secondary diabetes care in Southern Italy.

The following inclusion criteria were taken into account: T2DM, age ≥40 years, therapy with diet and/or oral hypoglycaemic agents during the first 3 years of the diagnosis of diabetes, persistent albuminuria ≥30 mg/24 h and moderate/severe diabetic retinopathy. Exclusion criteria were: type 1 diabetes, type 2 diabetes diagnosed below the age of 30 years or recently diagnosed in the last 3 months, insulin therapy during the first 3 years of diagnosis of the disease, severe liver or heart failure and known neoplastic or psychiatric disease.

Baseline information collected during the first visit, for screening and enrolment, included past medical history, with particular reference to major CV events (myocardial infarction and stroke), blood pressure measurement (calculated as a mean of three measurements taken in a sitting position after 10 min of rest), height and body weight as well as laboratory and therapeutic features. Laboratory tests were performed locally and included glycaemic, lipidic and renal function assessment. GFR was calculated by the four variable Modification of Diet in Renal Disease equation and albuminuria was measured on 24-h urine collection.

Active follow-up, with control visits planned every 6 months, was completed on 30 November 2013. In particular, adherence to therapy was assessed by questionnaire.

When a CV event was suspected, hospital records were collected to make the diagnosis according to the European Society of Cardiology. Death certificates and autopsy reports were used to establish the underlying cause of death and to identify CV deaths, through the ninth revision of the International Classification of Diseases.

### Characteristics of participants

We selected 564 patients free from CVD from this cohort.

169 of them were treated with statins (group A) while 395 of them were not on statins (group B). Both groups were homogeneous for age, BMI, HbA1c, blood pressure, lipid profile, albumin excretion rate (UAlb) and cardiovascular risk. Patients were followed up for 8 years, with control visits planned every 6 months. The choice of specific type and dose of statin was independently made by each individual physician for any patient, according to the personal clinical judgment. Moreover, the choice of introducing statin in the therapy was independently made by the physician, according to his/her perception and valuation of the CV risk in patients at primary CV prevention. The target for all patients was a LDL-cholesterol <100 mg/dl, according to previous ADA position statement [28].

### Outcomes

Primary outcome was total major adverse cardiovascular events (MACE), defined as CV death, nonfatal myocardial infarction and nonfatal stroke. Secondary outcomes were, separately, fatal and nonfatal CV events.

Twenty-one outpatient clinics in Campania region of Italy (a geographic area characterized by a homogeneous prevalence of type 2 diabetes) were randomly chosen among all the regional clinics. In the design and implementation process, any effort was made to ensure consistency across the 21 centers in terms of data specification, data collection tools, and methods and the analysis and reporting of results.

### Statistical analysis

Kaplan–Meier survival curves were used to compare cumulative probability of time free from MACE. The comparison between the two groups was performed using the log-rank test. To assess the independent effect of CV risk factors on the primary endpoint, hazard ratio, with 95 % confidence intervals (HR, 95 % CIs), was estimated by a Cox regression model with demographics (age and gender) and several potentially treatable risk factors (HbA1c, systolic blood pressure, total cholesterol, statin treatment) variables as covariates. Statistical significance was fixed at 0.05. Statistical analyses were performed using SPSS version 16.0 (SPSS Inc, Chicago, IL, USA) software package.

## Results

Patients had a mean age of 64.7 ± 8.8 years and were mostly females (55.1 %). They were mainly overweight (BMI 29.3 ± 4.6 kg/m^2^) and not at target according to ADA guidelines [15] for glycaemic control (HbA1c 7.4 ± 1.3 %) and systolic blood pressure (135.9 ± 13.2 mmHg). The diastolic blood pressure was 78.2 ± 7.3 mmHg, total cholesterol was 196 ± 41.2 mg/dl, LDL cholesterol was 118.7 ± 31.3 mg/dl and triglycerides were 149.3 ± 80.8 mg/dl. More than a quarter of the patients were smokers (25.7 %). Cardiovascular risk factors and drug treatments, except for statin therapy, were not statistically different among the group A and B at baseline. Detailed clinical characteristics of all subjects at baseline are summarized in Table 1.

**Table 1:** Demographic and clinical characteristics in patients at baseline in overall cohort and in groups A and B

	Overall (n 564)	Group A (n 169)	Group B (n 395)	p*
Gender (% m/% f)	44.9/55.1	43.5/56.5	45.1/54.9	0.22
Age (years)	64.7 ± 8.8	64.4 ± 8.4	65.2 ± 9.2	0.21
Duration of diabetes (years)	9.0 ± 4.1	9.2 ± 4.5	8.9 ± 4.0	0.19
Smoker (%)	25.7	26.4	25.2	0.18
BMI (kg/m^2^)	29.3 ± 4.6	29.6 ± 4.4	29.2. ± 4.7	0.32
HbA1c (%)	7.4 ± 1.3	7.4 ± 1.4	7.5 ± 1.0	0.26
SBP (mmHg)	135.9 ± 13.2	134.6 ± 15.6	136. ± 16.1	0.21
DBP (mmHg)	78.2 ± 7.3	77.5 ± 6.2	79.9 ± 7.0	0.27
Tot. cholesterol (mg/dl)	196 ± 41.2	198.4 ± 43.6	195.4 ± 40.5	0.19
HDL-C (mg/dl)	48.8 ± 11.9	48.9 ± 12,4	48.4 ± 11.7	0.25
LDL-C (mg/dl)	118.7 ± 31.3	119.5 ± 32.5	117.6 ± 30.9	0.16
Triglycerides (mg/dl)	149.3 ± 80.8	146.9 ± 83.4	150.1 ± 81.7	0.17
UAlb (mg/24 h)	143.1 ± 136.3	139.5 ± 140.4	144.4 ± 131.5	0.12
GFR (mL/min/1.73 m^2^)	66.4 ± 24.3	66.1 ± 28.1	67.2 ± 25.2	0.22
Anti-hypertensive drugs (%)	84.5 %	83.5	85.3	0.19
OHA (%)	67.7	66.5	68.3	0.20
Insulin (%)	29.4	30.1	28.8	0.18
Statins (%)	29.9	100	0	
Aspirin and/or other antiplatelet drug (%)	42.8	43.1	42.5	0.24

Values are mean ± SD, or percent

*GFR* estimated glomerular filtration rate using MDRD calculator, *HDL* high density lipoprotein, *LDL* low-density lipoprotein, *UAlb* urinary albumin excretion, *OHA* oral hypoglycaemic agents

* (p group A vs group B)

Most intriguingly, none of the patients was treated with a high-intensity statin therapy (e.g. atorvastatin 40–80 mg and rosuvastatin 20–40 mg daily) despite to the 2016 ADA position statement [15].

Therefore, the subjects of group A received one of the following: atorvastatin 10–20 mg, rosuvastatin 5–10 mg, simvastatin 20–40 mg, pravastatin 40–80 mg, Lovastatin 40 mg, fluvastatin 80 mg.

We registered 7 non CV deaths, 3 in group A (malignant neoplasms) and 4 in group B (3 malignant neoplasms and 1 accident).

Total MACE occurred in 32 patients (2.37 events/100 pts/year) from group A and 68 patients (2.15 events/100 pts/year) from group B. 13 fatal MACE (0.96 events/100 pts/year) and 19 nonfatal MACE (1.4 events/100 pts/year) occurred in group A. 30 fatal MACE (0.94 events/100 pts/year) and 38 nonfatal MACE (1.2 events/100 pts/year) occurred in group B. Notably, the distribution of MACE (fatal and not) was homogeneous among the centers and not clustered in just some of them.

**Fig. 1:**
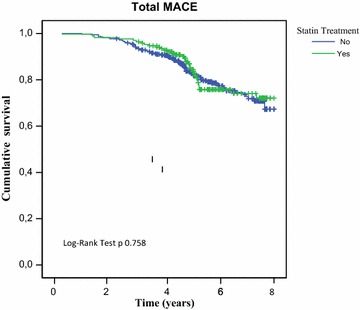
Kaplan–Meier estimates of total major adverse CV events in statin-treated group (*green line*) and non-statin-treated group (*blue line*), during the average follow-up of 8 years

The analysis of the Kaplan–Meier survival curves showed a not statistically significant difference in the incidence of total MACE in the two groups (p 0.758) (Fig. 1).

Moreover, no differences were observed between the two groups in the analysis of the secondary end points. In fact, the analysis of the Kaplan–Meier curves showed a not statistically significant difference in the incidence of both fatal and nonfatal MACE between the two groups (respectively p 0.474 and p 0.812).

To assess the independent effect of the CV risk factors on the primary end point, hazard ratio, with 95 % CIs, was estimated by a Cox regression model with demographics (gender) and several potentially treatable risk factors variables (smoking, statin therapy, HbA1c, systolic blood pressure, BMI and total cholesterol) as covariates (Table 2). HbA1c only showed a significant difference in the incidence of MACE between the two groups (HR 1.201, CI 1.041–1.387, p 0.012).

**Table 2:** Independent effect of the CV risk factors on the primary end point

	HR	CI 95 %		p
		Lower limit	Upper limit	
Age	1.017	0.994	1.039	0.145
Male gender	0.858	0.538	1.367	0.519
BMI (kg/m^2^)	1.014	0.972	1.057	0.524
HbA_1C_ (%)	1.201	1.041	1.387	0.012
Total cholesterol (mg/dl)	1.001	0.996	1.006	0.817
Systolic blood pressure (mmHg)	1.003	0.988	1.018	0.703
GFR (ml/min)	0.988	0.979	0.998	0.114
UAlb (mg/24 h)	1.000	1.000	1.001	0.502
Statin (yes/no)	0.759	0.496	1.161	0.204

**Fig. 2:**
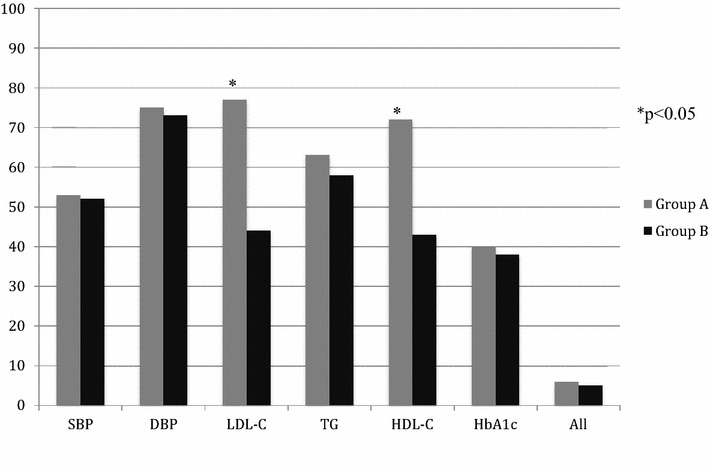
Rate of patients of both groups at target for major CV risk factors at ending of follow-up (8 years)

At the end of the follow up 6 % of subjects from group A and 5 % from group B were at target for major CV risk factors (BMI, glycaemic control, blood pressure, lipid profile and UAlb). Moreover more than one half of patients treated with statins were at target for LDL-cholesterol (118; 77 %) and HDL-cholesterol (110; 72 %), whereas 159 (44 %) and 155 (43 %) patients not treated with a statin where at target for LDL- and HDL-cholesterol respectively (Fig. 2).

In detail, at the end of 8 years of follow-up no significant difference between group A and B was shown in BMI (29.8 ± 4.5 vs 29.4 ± 4.9; p 0.35), HbA1c (7.8 ± 1.6 vs 7.9 ± 1.7; p 0.20), systolic blood pressure (137.6 ± 17.9 vs 138.4 ± 18.5; p 0.28), diastolic blood pressure (79.2 ± 7.3 vs 80.1 ± 6.9; p 0.25), triglycerides (156.2 ± 74.7 vs 161.1 ± 81.1; p 0.14), UAlb (148.3 ± 145.1 vs 158.8 ± 145.2; p 0.09) and GFR (55.3 ± 29.4 vs 56.4 ± 30.1; p 0.18). Conversely, a significant difference was observed between two groups in total cholesterol (179.8 ± 37.2 vs 206.2 ± 43.7; p 0.036), HDL-cholesterol (49.9 ± 11.9 vs 44.2 ± 14.5; p 0.028) and LDL-cholesterol (102.7 ± 28.9 vs 138.5 ± 38.3; p 0.011).

## Discussion

CVDs are the primary cause of mortality and morbidity globally and produce immense health and economic burdens [29]. This prospective and multicenter study shows the lack of CV protective effect of statin treatment in primary prevention in a T2DM population at very high CV risk, such as our cohort with clinical diagnosis of diabetic nephropathy (based on the concomitant presence of abnormal albuminuria and severe retinopathy). The CV outcome has never been evaluated for this high-risk population, but the cross-sectional phase of the NID-2 study pointed out that this category of patients showed a 10-year risk of CV events greater than 10 % [30]. In patients with T2DM and nephropathy the interaction between albuminuria and GFR is statistically significant, and it influences the risk for fatal and non-fatal CV events [30]. Moreover, both GFR and albuminuria have a great influence on CVD burden, independently of the classification system used for CKD in T2DM [31]. A recent large retrospective study on about 58,000 T2DM patients based on a UK primary care database confirms that all-cause mortality and the risk of cardiovascular events significantly increase with the decrease of GFR values [32]. Therefore, a careful management of GFR, albuminuria and CV risk factors represents a winning strategy to reduce the risk of fatal and non fatal CV events.

In this particular study we have only considered the population in primary CV prevention according to the above-mentioned inclusion criteria. It is very interesting to note that, according to 2014 ADA guidelines [28], the main aim for statin treatment was to gain the LDL-cholesterol target (<100 mg/dl in primary CV prevention, <70 mg/dl in secondary CV prevention), independently on the dose and the drug chosen.

The most recent ADA guidelines [15] recommend lifestyle modification to improve the lipid profile in patients with diabetes, and suggest an addition of high-intensity statin therapy for patients of all ages with diabetes and overt CVD (level A of evidence). Moreover, for primary CVD prevention diabetes patients should be treated with statins if they are aged over 40 years regardless of CV risk factors. In particular, for patients with diabetes aged 40–75 years with additional atherosclerotic cardiovascular disease risk factors (e.g. LDL cholesterol >100 mg/dl, high blood pressure, smoking, albuminuria, and family history of premature CVD), high-intensity statin and lifestyle therapy should be used (level B of evidence). According to the position statement of ADA, a high-intensity statin therapy (e.g. atorvastatin 40–80 mg and rosuvastatin 20–40 mg daily) reduces LDL cholesterol by >50 % [15].

These Guidelines were based on the observation of the effectiveness of specific doses of statins against placebo or other statins, rather than aiming at specific LDL cholesterol levels [33]. Thus, the ADA considers the patients with increased cardiovascular risk similarly to those with known CVD.

Notably, all patients of our cohort (group A) were treated with a moderate-intensity statin.

Because of the high rate of patients at target for LDL-cholesterol in group A, it seems evident that, in this observational study, all physicians followed the old “treat to target” method rather than the recent ADA position statement. Actually, we can state that, independently on the dose and kind of the statin used, physicians appropriately prescribed statins to the group A individuals.

Actually, evidence for the efficacy of statins is dominated by randomized controlled trials mostly focused on general population at high CV risk or on CVD secondary prevention [16–19]. However, there is still some controversy such the use of statins in diabetes patients for primary CVD prevention [20–23] and most of the trials are not specifically designed for selected categories of diabetic patients. All the statin clinical trials on diabetic populations examined patients with unspecific proteinuria. Our study is focused on type 2 diabetes complicated by diabetes nephropathy *stricto* sensu. A recent review [34], supporting the use of statins in primary CV prevention, showed that statins significantly reduce the risk of myocardial infarction, coronary death, coronary revascularisation and the risk of stroke in patients with documented diabetes at baseline irrespective of a prior history of vascular disease. However, the absolute risk for people with diabetes was probably affected by the entry criteria of the trials, so the observed absolute benefits of statin cannot be directly extended to any categories of people with diabetes but have to be evaluated case by case.

A meta-analysis pooled the data from eight randomized trials that compared statins with placebo in primary prevention in populations at increased CV risk and found that total mortality was not reduced by statins [20]. Similarly, another meta-analysis showed that treatment with lipid lowering drugs in primary prevention lasting 5–7 years reduced coronary heart disease events and mortality by about 30 % but their effect on all-cause mortality was not significant [35]. Moreover, a recent literature-based meta-analysis of 11 randomized controlled trials involving 65,229 participants did not find evidence for the benefit of statin therapy on all-cause mortality in a high-risk primary prevention set-up [36].

The most recently published Cochrane systematic review on statins for the primary prevention of CBD [37], included 18 randomised controlled trials (56,934 patients), dating from 1994 to 2008, that compare statins with usual care or placebo. All-cause mortality and fatal and non-fatal CVD events were reduced with the use of statins as it was the need for revascularisation (coronary artery bypass graft or angioplasty). Of these trials, only 4 included patients with diabetes: ASPEN, CARDS, MRC/BHF Heart Protection Study and CERDIA. The main outcome of CERDIA [38] was to determine the effect of statin therapy on the progression of carotid intima-media thickness, so only the other three have as main outcome the effects of statins in major vascular events.

The MRC/BHF Heart Protection Study is a large randomised placebo-controlled trial [39], conducted between 1994 and 1997, which recruited 2912 patients with diabetes (type 1 and type 2) without any diagnosed coronary or other occlusive arterial disease at study entry, of whom 50 % were allocated to simvastatin 40 mg. It showed a highly significant (nearly 30 %) proportional reduction of the first major vascular event in the group treated with simvastatin compared to the placebo. However, among the diabetic patients in CDV primary prevention, the absolute risk of major vascular events was influenced to a lesser extent by their initial concentrations of LDL-cholesterol as a 1.0 mmol/L reduction in LDL cholesterol would translate into avoidance of major vascular events during 5 years in about 3 % individuals compared to about 9 % in people with diabetes in CVD secondary prevention.

The West of Scotland Coronary Prevention Study (WOSCOPS) was the first trial to demonstrate a significant 31 % reduction in CV events for primary prevention in patients treated with statin therapy for 5 years [40]. The twenty-year follow-up of the WOSCOPS further suggested that treatment with a statin for 5 years might provide a persistent reduction in CVD mortality and hospitalizations, but no impact on stroke [41]. The AFCAPS study compared lovastatin with placebo for primary CV prevention in a population who had average total cholesterol and LDL-C and below-average HDL-C. After a 5 years follow up, treatment with lovastatin resulted in a 37 % reduction in the risk for first fatal or nonfatal acute major coronary events [42]. Compared to our results and those of the other trials on statin treatment in primary prevention, the WOSCOPS and the AFCAPS recruited a relatively younger population (mean age around 55–58 years) and only 1–2 % had a diagnosis of DM. Moreover the WOSCOPS recruited only males and with higher mean LDL cholesterol levels (192 mg/dl) and the AFCAPS excluded those managed with insulin.

Notably, although diabetic patients in our study had a mean BMI of 29.3 kg/m^2^, they did not experience a significant survival benefit from the prescription of statins before MI, unlike observed in overweight patients with diabetes elsewhere [43].

Statin treatment failed to show a positive effect on primary CV prevention in our cohort of high CV risk patients as shown by 18.9 % MACE, although partially related to the concomitant nephropathy. These findings are similar to those found in the Atorvastatin Study for Prevention of Coronary Heart Disease Endpoints in Non-Insulin-Dependent Diabetes Mellitus (ASPEN). In this study 2400 type 2 diabetic patients, of whom 1905 without prior myocardial infarction, were randomized to atorvastatin or placebo and were followed up for 4 years. Primary composite end point (CV death, nonfatal myocardial infarction, nonfatal stroke, recanalization, coronary artery bypass grafting, resuscitated cardiac arrest, or worsening or unstable angina requiring hospitalization) was not significantly different between the two groups [44]. These results are further supported by other RCTs, such as the CARDS study which enrolled T2DM patients aged 40–75 years with no documented previous history of cardiovascular disease and at least an additional CV risk factor (retinopathy, albuminuria, current smoking or hypertension). After 3.9 years of follow-up, atorvastatin treatment showed a significant reduction of MACE, but didn’t record a significant fall in all-cause mortality [45]. Notably, in these two studies atorvastatin was used at high-intensity doses.

In the Anglo-Scandinavian Cardiac Outcomes Trial (ASCOT) over 19,000 hypertensive patients and with at least three additional CV risk factors were randomized to receive amlodipine or atenolol. The lipid-lowering arm **(**LLA**)** of the trial randomized a subgroup of patients to additional treatment with atorvastatin or placebo, of whom 2532 were classified as having type 2 diabetes. After 3 years of follow-up there was a significant reduction in MACEs among the patients allocated on atorvastatin, although these reductions were not statistically significant in the DM subgroup [46]. Moreover the 11-year mortality follow-up of the lipid-lowering arm in the UK confirmed any significant reduction of CV deaths [47].

PROSPER was a controlled, randomized study involving around 6000 patients aged over 70 years, with a history of or risk factors for CVD. During the 3 years of follow-up, pravastatin reduced the risk of fatal and non-fatal CVD, but did not affect the risk of stroke and did not demonstrate a reduction in mortality [48]. Moreover the extended follow up over 8.6 years found no evidence that treatment of older high-risk subjects with pravastatin for several years prolonged life expectancy as it failed to show any reduction in stroke or all-cause mortality [49]. The Antihypertensive and Lipid-Lowering Treatment to Prevent Heart Attack Trial **(**ALLHAT-LLT**)** was a multicenter study conducted in 10,355 hypertensive patients with mean age of 66 years, of whom 14 % had a history of CVD and 25 % had T2DM. After a follow up of 3.3 years, reduction in all-cause mortality or CVD was not significant in the group treated with Pravastatin compared with usual care [50].

Our study shared similar baseline characteristics of the population recruited in the above trials, especially with regard to the glycaemic control (HbA1c < 10 %), blood pressure (<140/80 mmHg), BMI (<30 kg/m^2^) and LDL (<130 mg/dl). We had a higher percentage of smokers (26 %) and females (55 %). Moreover we recruited patients with clinically detected diabetic nephropathy. The CARDS was the only study that mentioned 17 % of their diabetic population had micro- or macroalbuminuria. The ASCOT and ALLHAT included hypertensive patients whereas the PROSPER recruited an older population (mean age 75 years). Compared to our cohort, the Heart Protection Study recruited patients with both type 1 and type 2 diabetes and shorter disease duration (<5 years), better glycaemic control (mean HbA1c 7 %) and better renal function (mean baseline creatinine 88 µmol/L).

From these studies appeared that the impact of statins on CV outcomes in diabetic patients is different between primary and secondary prevention. In particular the findings from studies in primary prevention were not obtained by high-intensity statin. Therefore, the recent ADA position statement seems to be not supported by experimental evidence (level B).

More studies are required about unknown actions and overall actions of statins, regarding to potential side effects of statin use for short-term [51] and, particularly, extended periods [52].

## Conclusions

Our study originally showed:

1. The consolidated habit of physicians to use as goal of therapy the achievement of the LDL-C target;
2. The difficulties in making as targets all risk factors and, hence, the failure to control the residual risk;
3. Lack of effectiveness of traditional therapy with a statin alone on primary prevention in high-risk individuals.

The main methodological limitation of this study is its observational nature. Moreover, since this study is not a RCT, the sample size was not preliminary measured, and in particular it has insufficient statistical power. On the other hand, the cohort was recruited from 21 centers and the mean follow up was at 8 years. Therefore, in this investigation a population at high CV risk was regularly followed for long time by expert specialists at a large number of diabetic clinics according to good medical practice. In this way, we trust that these conditions were modeled on real life and our findings were not “doped” by the trial effect occurring in RCTs. In particular we observed a great dichotomy between the new guidelines and the medical practice in patients at high risk in primary CV prevention.

Our study shows the inefficacy of statin therapy for primary CVD prevention in patients with T2DM and clinically detected diabetic nephropathy. These findings could be due to the moderate-intensity statin therapy used by physicians. Actually, physicians choose type and dose of statin using a “treat to target” method referred to LDL-cholesterol, without respecting the recent position statement of ADA. This therapeutic choice could probably explain the lack of difference in the incidence of total MACE between the two groups, despite the achievement of the “classic” targets for LDL- and HDL-cholesterol levels in a higher percentage of the statin treated group. Additionally, all the other major CV risk factors were at target in less than 10 % of patients regardless of statin treatment. These findings confirm that although statins are effective for the reduction of the incidence of fatal and non-fatal MI, the residual global CV risk remains high, as people with diabetes have multiple modifiable risk factors not influenced by this class of medications. Current guidelines on statin treatment for primary CV prevention do not apply to the “real life” high risk diabetic population of our study and, as such, these guidelines would need a strong implementation in clinical practice. A long-term targeted multifactorial intervention trial has to be considered in order to try to reduce significantly the residual risk in diabetic population in primary CV prevention, because this is a major challenge for the clinicians as well as national health systems.

## Abbreviations

CVD: cardiovascular disease
DM: diabetes mellitus
MACE: major adverse cardiovascular events
MI: myocardial infarction
ADA: American Diabetes Association
GFR: glomerular filtration rate using
HDL: high density lipoprotein
LDL: low-density lipoprotein
UAlb: urinary albumin excretion
OHA: oral hypoglycaemic agents
